# Adjusting selection bias in German health insurance records for regional prevalence estimation

**DOI:** 10.1186/s12963-019-0189-5

**Published:** 2019-08-27

**Authors:** Ralf Thomas Münnich, Jan Pablo Burgard, Joscha Krause

**Affiliations:** Institution-Department of Economic and Social Statistics, Trier University, Universitätsring 15, 54286 Trier, Germany

**Keywords:** Health insurance records, Selection bias, Multi-level modelling, Regional prevalence estimation, Diabetes mellitus

## Abstract

**Background:**

Regional prevalence estimation requires epidemiologic data with substantial local detail. National health surveys may lack in sufficient local observations due to limited resources. Therefore, corresponding prevalence estimates may not capture regional morbidity patterns with the necessary accuracy. Health insurance records represent alternative data sources for this purpose. Fund-specific member populations have more local observations than surveys, which benefits regional prevalence estimation. However, due to national insurance market regulations, insurance membership can be informative for morbidity. Regional fund-specific prevalence proportions are selective in the sense that the morbidity structure of a fund’s member population cannot be extrapolated to the national population. This implies a selection bias that marks a major obstacle for statistical inference. We provide a methodology to adjust fund-specific selectivity and perform regional prevalence estimation from health insurance records. The methodology is applied to estimate regional cohort-referenced diabetes mellitus type 2 prevalence in Germany.

**Methods:**

Records of the German Public Health Insurance Company from 2014 and Diagnosis-Related Group Statistics data are combined within a benchmarked multi-level model. The fund-specific selectivity is adjusted in a two-step procedure. Firstly, the conditional expectation of the insurance company’s regional prevalence given related inpatient diagnosis frequencies of its members is quantified. Secondly, the regional prevalence is estimated by extrapolating the conditional expectation using corresponding inpatient diagnosis frequencies of the Diagnosis-Related Group Statistics as benchmarks. Model assumptions are validated via Monte Carlo simulation. Variable selection is performed via multivariate methods. The optimal model fit is determined by analysis of variance. 95% confidence intervals for the estimates are constructed via semiparametric bootstrapping.

**Results:**

The national diabetes mellitus type 2 prevalence is estimated at 8.70% with a 95% confidence interval of [8.48%, 9.35%]. This indicates an adjustment of the original fund-specific prevalence from − 32.79 to − 25.93%. The estimated disease distribution shows significant morbidity differences between regions, especially between eastern and western Germany. However, the cohort-referenced estimates suggest that these differences can be partially explained by regional demography.

**Conclusions:**

The proposed methodology allows regional prevalence estimation in remarkable detail despite fund-specific selectivity. This enhances and encourages the use of health insurance records for future epidemiologic studies.

## Background

Regional prevalence estimation has gained considerable attention in epidemiologic research over the past years [1–5]. Policymakers and health care providers need reliable information on regional disease distributions to plan comprehensive health programmes, like, e.g. the EU Health Programme 2014–2020 in the European Union [6]. Depending on the disease of interest and national privacy regulations, corresponding figures may not be recorded in registries and thus must be estimated. This requires epidemiologic data with substantial local detail to ensure stable results on regional levels. Large-scale surveys, such as NHANES in the USA, represent solid data bases for such a purpose. However, if a survey of this size is not available, regional prevalence estimation suffers from low accuracy. Using smaller surveys that lack in sufficient local observations has to be viewed critically, as non-random local heterogeneity in health outcomes has been found in several studies [7–9]. Thus, using smaller surveys for regional prevalence estimation encloses the risk of missing unobserved morbidity patterns between regions. If multiple surveys on similar topics are available, they can be combined to achieve better results, as demonstrated by [3]. However, this case is not considered within this study.

We discuss health insurance (HI) records as a substitute for epidemiologic data obtained from large surveys to perform regional prevalence estimation. They provide four significant benefits. Firstly, the records are resource-efficient, as they do not have to be collected in expensive surveys. Secondly, the member population of a HI fund is typically large and has much more local observations than a survey sample. This encloses an information advantage on the local level that is crucial for capturing regional morbidity patterns. Thirdly, HI records contain a vast variety of health figures, like diagnosis frequencies, required medicine, or medical treatments. They are collected whenever a member faces an insurance-relevant situation. This allows for more flexibility in the analysis compared to a fixed set of survey observations. And finally, HI records can be used in aggregated form to obtain prevalence estimates, as will be shown in this study. Survey-based prevalence estimation typically requires micro data, which is particularly sensitive in the health context.

Besides these benefits, the utility of HI records for prevalence estimation is frequently questioned. An important objection is that HI membership can be informative for morbidity. Depending on national HI market regulations, there are several selection mechanisms associated with HI membership. As a result, the morbidity structure of a particular HI fund, especially in terms of regional prevalence proportions within its member population, does not necessarily apply to the national population [10, 11]. We refer to this as fund-specific selectivity. A salient example for fund-specific selectivity is Germany for several reasons. In general, HI membership is mandatory in Germany. However, the German HI market is characterized by a rather unique distinction between statutory HI and private HI. Roughly 90% of the citizens are members of statutory HI funds and contribute a fixed proportion of their monthly salary as premium. The remaining 10% are self-employed or citizens with a salary above a certain threshold. They are allowed to drop out of the statutory system and join a private HI fund instead. In the majority of cases, private HI is more expensive due to risk-based premiums, but provides better HI benefits. As a consequence, the distinction between statutory and private HI is linked to selectivity in terms of income and socioeconomic status [12, 13].

Secondly, German HI funds had been associated with specific clientele of the economy from the late nineteenth century until 1996. Thus, throughout the majority of German HI history, HI membership was determined by the branch of the economy a citizen was employed in. This caused individual HI funds to cover industry-dependent morbidity structures. Although this regulation is now suspended, job-related morbidity differences between HI member populations still exist to some degree [10, 14]. And finally, there are considerable differences in health outcome between regions, for example when comparing the former territories of the Federal Republic of Germany and the German Democratic Republic (western and eastern Germany) [9]. Since some HI funds only cover regional populations, this is yet another source of selectivity. Subsequently, HI membership in Germany is informative for morbidity and there are substantial prevalence differences between member populations of German HI funds [9, 15, 16]. Therefore, when performing regional prevalence estimation from HI records, the researcher encounters a selection bias that must be adjusted to obtain accurate results. While it is plausible to conclude that not every aspect of the selectivity can be accounted for, it is nevertheless possible to adjust fund-specific regional prevalence proportions and extrapolate them to the national population by using suitable auxiliary data.

We propose a methodology to account for the fund-specific selectivity of an individual HI fund’s morbidity structure to perform regional prevalence estimation. HI records and data of Diagnosis-Related Group Statistics (DRG Statistics) are combined within a benchmarked multi-level regression model that corrects the bias and predicts highly detailed prevalence proportions in a two-step procedure. Semiparametric bootstrapping is used to construct 95% confidence intervals for the estimates. Variable selection is performed via multivariate methods. The optimal model fit is determined by analysis of variance (ANOVA). The model assumptions are validated by Monte Carlo simulation and cross-validation.

The methodology is applied to estimate regional diabetes mellitus type 2 prevalence in Germany. This disease is chosen for two reasons. Firstly, it is known to be asymmetrically distributed among German HI funds due to its close relation to socioeconomic status [10]. Secondly, there are large differences in diabetes mellitus type 2 prevalence on regional levels, as shown by [17]. We use aggregated records of the German Public Health Insurance Company (AOK) from 2014 to estimate age- and sex-referenced prevalence proportions for all German districts. Using the AOK member population as initial data basis represents a valid test for the proposed methodology, as it is known to have a fairly unique morbidity structure that differs from the national population. Prevalence of overweight and smoking is higher, whereas socioeconomic status is on average lower compared to members of other HI funds [13]. Further, AOK members have the highest diabetes mellitus morbidity among all German HI funds [15].

## Methods

### Data

#### AOK member population

The first data basis considered for this study is the HI records of the AOK member population from 2014. Here, AOK members are defined as all citizens that have been enrolled for at least 1 day in 2014. Age and residence of the members were defined according to the latest version of the data within the AOK member records in the corresponding report year. The AOK covered about 24.16 million members in 2014, which was roughly 35% of all statutory HI members. From this population, we extract aggregated records only, no person-specific data is used. The aggregates are referred to as cells and represent cross combinations of administrative districts, age groups and sex. Germany consists of 402 districts, the age groups are constructed as I: 34 and younger, II: 35–49, III: 50–69 as well as IV: 70 and older. The total number of cells is 3216.

From the AOK member population, the total number of AOK members per cell is retrieved to obtain information on the regional demography. Further, the number of AOK members that are diagnosed with diabetes mellitus type 2 per cell is extracted. The members concerned are identified by an intersectoral disease profile within the AOK records. See Appendix 1 for more details. In addition to that, inpatient diagnosis frequencies of AOK members per cell are gathered. The diagnoses are registered on ICD-3 level, both primary and secondary diagnoses are included. Note that the data does not contain information on how many AOK members were treated with a given diagnosis in a German hospital, but on how often a given diagnosis associated with AOK members was treated in 2014 (count of cases, not persons). The records are referenced by age group, sex and residence of the AOK members. This allows an exact matching of the inpatient diagnosis frequencies to the demographic and prevalence data.

#### DRG Statistics

The second data basis is the DRG Statistics from 2014 published by the German Federal Statistical Office [18]. The data contains frequencies of inpatient main and secondary diagnoses on the ICD-3 level. Analogous to the hospital-related data set generated from the AOK member population, it provides information on how often a given diagnosis was treated in German hospitals with respect to age group, sex and residence of the patients. The data source is constructed as an annual census. All German hospitals are obliged to provide the corresponding information to a certain reference date. Note that the records are aggregated on the cell level, no person-specific data is required. The aggregated records allow an exact matching to the records of the AOK member population. However, the data does not only include AOK members, but all German citizens that have been hospitalized in a given year. Therefore, the records of the DRG Statistics represent a national population analogue to the inpatient diagnose frequencies of the AOK member population.

#### Population statistics

The third data basis is the population statistics from 2014 published by the German Federal Statistical Office [19]. These records contain information on how many citizens live within an individual cell in the national population. The data corresponds to the population status on 31 December 2014. The data represents a national population analogue to the demographic data of the AOK member population.

#### Data usage

These three data bases are now combined within a statistical framework that allows regional prevalence estimation despite fund-specific selectivity. The general idea is to adjust the resulting selection bias in a two-step procedure. Firstly, the conditional expectation of the regional AOK prevalence given regional demography and suitable comorbidity variables of the AOK members is quantified. The suitable comorbidity variables are obtained by means of variable selection from the inpatient main and secondary diagnosis frequencies of the AOK member population. Secondly, the conditional expectation is extrapolated to the national population by using the population statistics and the corresponding DRG Statistics analogues to the selected comorbidity variables as benchmarks. If regional demography and comorbidity variables enclose sufficient explanatory power for the disease of interest, then the structural morbidity differences between the populations can be explained by a suitable regression model. This model then allows a bias-adjusted regional estimation of the national population prevalence.

### Statistical framework

Note that although regional prevalence estimation is discussed in a specific data setting within this paper, the subsequent statistical framework is formulated such that it can also be applied to others. Assume the member population of a single HI fund and all its health-related information are available as the initial data source. Let *U* denote the national population of size *N*, whose prevalence of a given disease shall be estimated locally and with respect to age and sex. *U* can be expressed as the unification of members $\overset{\sim }{U}$ and non-members $U\backslash \left\{\overset{\sim }{U}\right\}$ of the HI fund. Within the model, *U* can be additionally structured into three hierarchical levels of aggregation:

- ➢ Level 1: regions *r* of size *N*_*r*_ (*r* = 1, …, *R*),
- ➢ Level 2: districts *d* of size *N*_*rd*_ (*d* = 1, …, *D*_*r*_),
- ➢ Level 3: cells *c* of size ${N}_{rd}^c$ (*c* = 1, …, *C*), representing age and sex combinations.

The total number of cells *C* within a district is determined by the number of age groups the researcher wants prevalence estimates for. The cell sizes ${N}_{rd}^c$ as well as the share of members ${\overset{\sim }{N}}_{rd}^c/{N}_{rd}^c$ within each cell are known from HI records and population statistics. For simplicity, it is assumed that every cell has a non-empty subset of members (${\overset{\sim }{N}}_{rd}^c>0\forall r,d,c$). This assumption will be further discussed in the “Discussion” section. The objective is to estimate the cell level prevalence for the national population *U*. Let ${y}_{rd}^c$ be the unknown number of diseased citizens in cell *c* of district *d* in region *r*. From the HI records, the number of diseased members $\tilde{y}_{r}d^c\le {y}_{rd}^c$ within each cell is known. Due to the fund-specific selectivity in terms of morbidity, a prevalence scaling of $\tilde{y}_{r}d^c$ that is achieved by accounting for observable demographic differences between the members and the national population on cell level obtains biased estimates of ${y}_{rd}^c$:

1 $$ E\left(\frac{N_{rd}^c}{{\overset{\sim }{N}}_{rd}^c}\tilde{y}_{r}d^c\right)\ne {y}_{rd}^c \forall r,d,c, $$

where ${N}_{rd}^c/{\overset{\sim }{N}}_{rd}^c$ denotes the demographic scaling factor. Accordingly, there are systematic differences between the prevalence of the member population and the national population on cell level. This marks the baseline problem of our contribution.

Expression (1) implies that estimating the unknown ${y}_{rd}^c$ from the known $\tilde{y}_{r}d^c$ by exclusively considering age and sex is invalid. Subsequently, an additional data source that further explains the prevalence differences between the member population and the national population on cell level is required for unbiased estimates. If a data source explaining all differences between the populations exists, then $\tilde{y}_{r}d^c$ can easily be adjusted in order to quantify ${y}_{rd}^c$. However, given potential privacy issues mentioned earlier, it is unlikely that such information can be found in a single data set. Therefore, the required information must be retrieved from multiple data sources, as described hereafter.

First, a data set containing comorbidity variables with regard to the disease of interest has to be found for the national population. As explained previously, inpatient main and secondary diagnosis frequencies from the German DRG Statistics are used for this purpose within this study. The corresponding records are available on the cell level we seek prevalence estimates for. Next, a member analogue of this data has to be generated for the member population using the HI records. Given the detailed information the HI collects on its members, this represents no further issue. Note that the latter data set exclusively corresponds to the member population, but encloses the same variables on cell level as the records of the DRG Statistics. We refer to these two data sets as national population and member population auxiliary data.

The auxiliary data is likely to contain some information that is irrelevant for the disease of interest. Therefore, some form of variable selection must be performed in order to preserve degrees of freedom, keep the variance of the model parameter estimates on acceptable levels and thus ensure stable prevalence estimates. The choice of a variable selection criterion should be determined by the disease of interest. If it is a disease that is likely to have strong predictors within the auxiliary data, then correlation analysis or information criteria (e.g. AIC, BIC) may be appropriate. An example for such a case is a disease that usually requires hospitalization, while the auxiliary data contains inpatient records. However, for a disease that has no obvious predictors in the auxiliary data, the relevant comorbidity variables may be enclosed as latent variables in the data. These latent variables could then be retrieved by multivariate methods like principal component analysis or cluster analysis. Accordingly, also diseases not directly linked to the auxiliary data can be estimated.

Once a set of predictors has been identified within the HI auxiliary data, this exact set must be isolated in the national population auxiliary data as well. Let ***X*** denote a ($C\cdotp {\sum}_{r=1}^R{D}_r\times P$)-matrix of selected national population auxiliary variables on the cell level. Let $\overset{\sim }{\boldsymbol{X}}$ be the member analogue matrix of ***X*** with the same dimensions. Further, let ${\boldsymbol{x}}_{rd}^c$ and ${\overset{\sim }{\boldsymbol{x}}}_{rd}^c$ be (1 × *P*)-vectors representing the corresponding auxiliary data for a specific cell. If the auxiliary data can sufficiently explain the prevalence differences between the two populations, then their conditional expected cell level prevalences have the relation:

2 $$ E\left[{y}_{rd}^c|{N}_{rd}^c,{x}_{rd}^c=\left({x}_{rd}^{c1},\dots, {x}_{rd}^{cP}\right)\right]=E\left[\tilde{y}_{r}d^c\Big\Vert {\overset{\sim }{N}}_{rd}^c,\tilde{x}_{r}d^c=\left(\tilde{x}_{r}d^{c1},\dots, \tilde{x}_{r}d^{cP}\right)\right]. $$

Hence, after conditioning on the different cell sizes of the populations and the auxiliary data, there are no systematic prevalence differences between national population and member population in expectation anymore. If this holds, the information advantage in the HI records can be used to estimate the conditional expectation of $\tilde{y}_{r}d^c$ given ${\overset{\sim }{\boldsymbol{x}}}_{rd}^c$ very precisely. Then, ${y}_{rd}^c$ can be estimated using ${\boldsymbol{x}}_{rd}^c$ as benchmark. As Eq. (2) marks a crucial assumption for the model introduced hereafter, its validity has to be evaluated carefully. In particular, two conditions required for inference have to be established. Firstly, predicting regional prevalence proportions from inpatient diagnosis frequencies must be possible. This is validated by a Monte Carlo simulation using the HI records. Resamples are drawn from the member population under several scenarios that mimic the structural morbidity differences between the members and the national population. The regional prevalence proportions of the resample populations are estimated from the corresponding inpatient diagnosis frequencies by extrapolating the conditional expectation of the member prevalence given the member auxiliary data. If the results are unbiased in expectation, inferring the prevalence proportions from the hospital case numbers is valid.

Secondly, extrapolating the members’ prevalence proportions after morbidity adjustment to the national population must be valid. This is checked via cross-validation within the hospital data. The inpatient diagnosis frequencies of the disease of interest on the cell level are used as a proxy for the real prevalence. Note that the inpatient diagnosis frequencies are known for both the member and the national population. The conditional expectation of the member’s inpatient frequency of the disease of interest given closely related inpatient diagnosis frequencies is extrapolated to the national population using the national population inpatient diagnose frequencies as benchmarks. If the results are in expectation equal to the real national population inpatient frequency of the diagnosis of interest, this aspect of inference is valid as well. Within this study, both conditions could be successfully established.

### Model

The principle of equality in conditional expectations is now used to model the cell level prevalence of the national population. We consider a linear mixed model [20] with a multi-level structure:

3 $$ {\boldsymbol{y}}_{rd}={\boldsymbol{X}}_{rd}{\boldsymbol{\beta}}_r+{\boldsymbol{Z}}_{rd}{\boldsymbol{b}}_{rd}+{\boldsymbol{e}}_{rd}, $$

with ***y***_*rd*_ as (*C* × 1)-vector of cell level prevalence figures, ***X***_*rd*_ as (*C* × *P*)-matrix of fixed effect covariates and ***β***_*r*_ as (*P* × 1)-vector of fixed effects. ***Z***_*rd*_ is a (*C* × *Q*)-matrix of random effect covariates, while ***b***_*rd*_ ∼ *N*(**0**, **Ψ**) is a (*Q* × 1)-vector of random effects with some positive-definite variance-covariance matrix **Ψ**. ${\boldsymbol{e}}_{rd}\sim N\left(\mathbf{0},{\sigma}_r^2{\boldsymbol{I}}_C\right)$ is a (*C* × 1)-vector of cell-specific random errors with regional variance parameter ${\sigma}_r^2$. We further assume *Cov*(***b***_*rd*_, ***e***_*rd*_) = **0**. Note the different levels of the model components. The fixed effects in ***β***_*r*_ are allowed to vary systematically over regions, while the random effects in ***b***_*rd*_ vary randomly over districts. With the random errors in ***e***_*rd*_ varying over cells, variation is captured on all levels of aggregation. This enables accounting for the spatial heterogeneity discussed earlier. The model is fitted on the regional level; thus, a set of *R* sub-models is obtained:

4 $$ {\boldsymbol{y}}_r={\boldsymbol{X}}_r{\boldsymbol{\beta}}_r+{\boldsymbol{Z}}_r{\boldsymbol{b}}_r+{\boldsymbol{e}}_r\kern2em \forall r=1,\dots, R, $$

where ${\boldsymbol{y}}_r=\left({\boldsymbol{y}}_{r1},\dots, {\boldsymbol{y}}_{r{D}_r}\right)^{\prime }$, ${\boldsymbol{X}}_r=\left({\boldsymbol{X}}_{r1},\dots, {\boldsymbol{X}}_{r{D}_r}\right)^{\prime }$ and ${\boldsymbol{Z}}_r=\mathit{\operatorname{diag}}\left({\boldsymbol{Z}}_{r1},\dots, {\boldsymbol{Z}}_{r{D}_r}\right)$. Note that ***b***_*r*_ ∼ *N*(**0**, **Ψ**_*r*_) with **Ψ**_*r*_ = blockdiag(**Ψ**) and ${\boldsymbol{e}}_r\sim N\left(\mathbf{0},{\sigma}_r^2{\boldsymbol{I}}_{C{D}_r}\right)$. In order to estimate the model parameters, a sufficient number of observations from the endogenous variable is required. However, since the national population prevalence is not observed on cell level, the member prevalence $\tilde{y}_{r}d^c$ is used as a proxy. This is valid due to the equality of conditional expectations established in (2). Hence, as the functional relation between the prevalence and the auxiliary data is the same for both populations, we are able to estimate the parameters required for modelling the national population prevalence from the HI records. The parameter estimates are, thus, given by [21]:

5 $$ {\hat{\boldsymbol{\beta}}}_r={\left({\overset{\sim }{\boldsymbol{X}}}_r^{\prime }{\hat{\boldsymbol{V}}}_r^{-\mathbf{1}}{\overset{\sim }{\boldsymbol{X}}}_r\right)}^{-\mathbf{1}}{\overset{\sim }{\boldsymbol{X}}}_r^{\prime }{\hat{\boldsymbol{V}}}_r^{-\mathbf{1}}{\overset{\sim }{\boldsymbol{y}}}_r,\kern2.5em {\hat{\boldsymbol{b}}}_r={\hat{\boldsymbol{\Psi}}}_r{\boldsymbol{Z}}_r^{\prime }{\hat{\boldsymbol{V}}}_r^{-\mathbf{1}}\left({\overset{\sim }{\boldsymbol{y}}}_r-{\overset{\sim }{\boldsymbol{X}}}_r{\hat{\boldsymbol{\beta}}}_r\right) $$

with ${\overset{\sim }{\boldsymbol{X}}}_r$ and ${\overset{\sim }{\boldsymbol{y}}}_r$ as member analogues of ***X***_*r*_ and ***y***_*r*_. ${\hat{\boldsymbol{V}}}_r$ is an estimate of $\boldsymbol{V}={\boldsymbol{Z}}_r{\boldsymbol{\Psi}}_r{\boldsymbol{Z}}_r^{\prime }+{\sigma}_r^2{\boldsymbol{I}}_{C{D}_r}$ where the variance parameters are estimated via restricted maximum likelihood. Afterwards, the estimated model parameters are combined with the total population auxiliary data as benchmarks in order to estimate the cell level prevalence of the national population according to

6 $$ {\hat{y}}_{rd}^c={x}_{rd}^{c\prime }{\hat{\beta}}_r+{z}_{rd}^{c\prime }{\hat{b}}_{rd}, $$

where ${\boldsymbol{x}}_{rd}^c,{\boldsymbol{z}}_{rd}^c$ are the elements of ***X***_*r*_, ***Z***_*r*_ corresponding to a given cell. The resulting estimates then have the following regional distribution under the model assumptions:

7 $$ {\hat{\boldsymbol{y}}}_r\sim N\left({X}_r{\hat{\beta}}_r,{Z}_r{\hat{\Psi}}_r{Z}_r^{\prime }+{\hat{\sigma}}_r^2{I}_{C{D}_r}\right). $$

## Results

### Empirical specification

The methodology is applied to estimate the diabetes mellitus type 2 prevalence for the national population of Germany in 2014 on cell level. As stated before, cells represent cross combinations of administrative districts, age groups and sex. Note that Germany consists of 16 federal states which correspond to the regions within the “Methods” section. With regard to the region-specific model fit (4), the fixed effects thus vary between federal states. However, in view of potential other applications, it is important to mention that the number of regions (and thus sub-models) should not only depend on the population structure, but also on model complexity. Sophisticated model specifications and random effect designs are usually costly in terms of degrees of freedom. Hence, depending on the complexity, it may be useful to combine geographic territories in order to have more cells for model parameter estimation per region. Accordingly, there is a trade-off between accounting for local heterogeneity and model parameter variance.

A set of 10 diagnoses from the DRG Statistics and demographic information, such as cell size, age group affiliation and sex, are taken as fixed effects. The 10 diagnoses were chosen via correlation analysis of the AOK member diabetes prevalence and the inpatient diagnosis frequencies of the AOK members. Note that this analysis was performed for each region individually in order to account for systematic heterogeneity. We provided the 10 most frequently elected diagnoses subsequently in alphabetical order:

- E11 (secondary): Type 2 diabetes mellitus
- E78 (secondary): Disorders of lipoprotein metabolism and other lipidemias
- E87 (secondary): Other disorders of fluid, electrolyte and acid-base balance
- G81 (secondary): Hemiplegia and hemiparesis
- I10: (secondary) Essential (primary) hypertension
- I48 (main): Atrial fibrillation and flutter
- I63 (main): Cerebral infarction
- K29 (secondary): Gastritis and duodenitis
- M16 (main): Osteoarthritis of the hip
- T81 (secondary): Complications of procedures, not elsewhere classified

Note further that we are not allowed to provide any of the corresponding fixed effect sizes (regional beta values) due to disclosure restrictions. Random effects for several predictors are specified on the district level. The inclusion of multi-level interaction terms is evaluated via ANOVA and the conditional Akaike information criterion proposed by [22], but using the stronger penalty for additional parameters of the Bayesian information criterion due to limited degrees of freedom. Semiparametric bootstrapping with 1000 replicates is applied to construct 95% confidence intervals for the estimates according to [23].

### Prevalence estimates

The estimated nationwide diabetes prevalence of the national population is 8.70% with a 95% confidence interval [8.48%, 9.35%]. This implies a relative adjustment of the nationwide AOK member population prevalence from − 32.79 to − 25.93% by the model. Note that these figures refer to the crude prevalence only. However, as the estimates are on cell level, the crude prevalence can be depicted for any cohort individually and thus allows for age and sex adjustment.

**Fig. 1:**
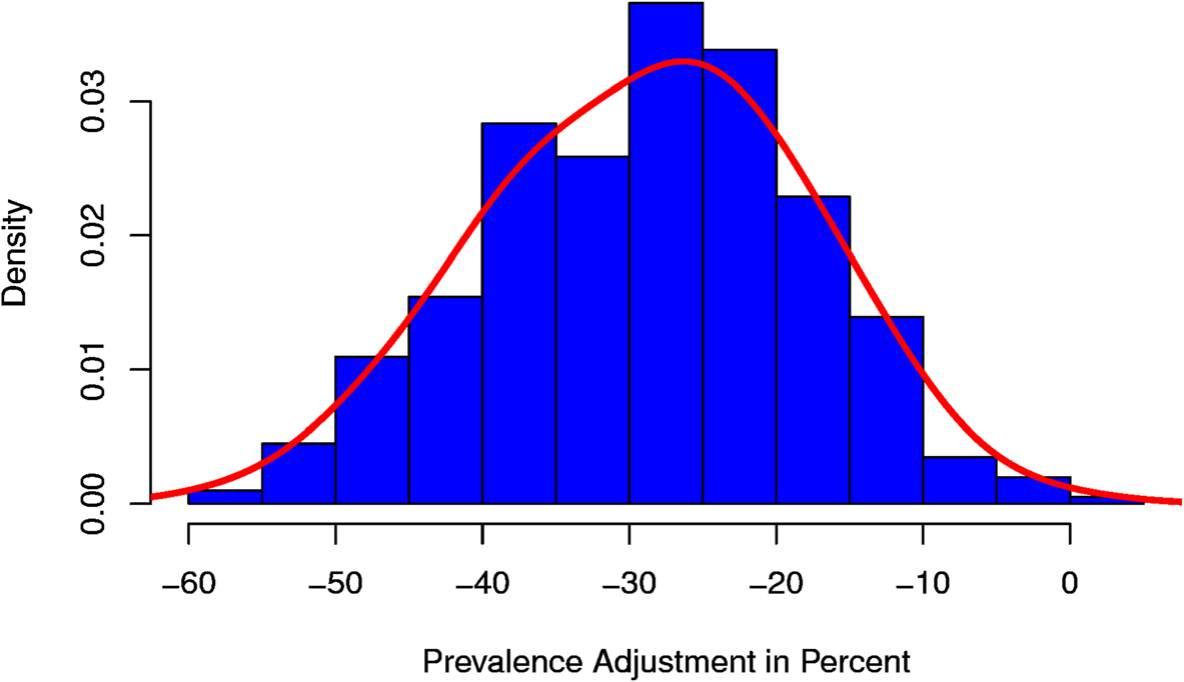
Relative prevalence adjustment on district level

Figure 1 shows the distribution of the relative prevalence adjustments from the AOK member population to the national population. The results are displayed on the district level. As can be seen, the majority of prevalence adjustments is negative. The overall distribution ranges from − 59.58 to 1.84% with mean − 28.06%.

**Fig. 2:**
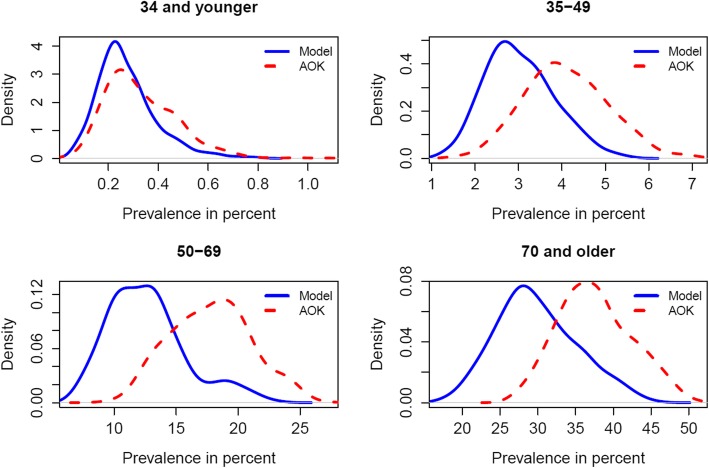
Comparison of age-specific prevalence densities

In Fig. 2, the estimated national population prevalence is compared to the AOK member population prevalence on the district level with respect to the four age groups defined in the “Methods” section. The graph suggests that the morbidity differences between the two populations are not uniform over all cohorts. The relative age-specific prevalence adjustments are:

- Age group I, − 20.37%
- Age group II, − 28.46%
- Age group III, − 30.83%
- Age group IV, − 22.27%

While prevalence densities in age groups I and IV are comparatively similar, the differences in age groups II and III are more significant. Accordingly, the morbidity differences between the AOK member population and the national population are most evident in cohorts that enclose the majority of the working population.

**Fig. 3:**
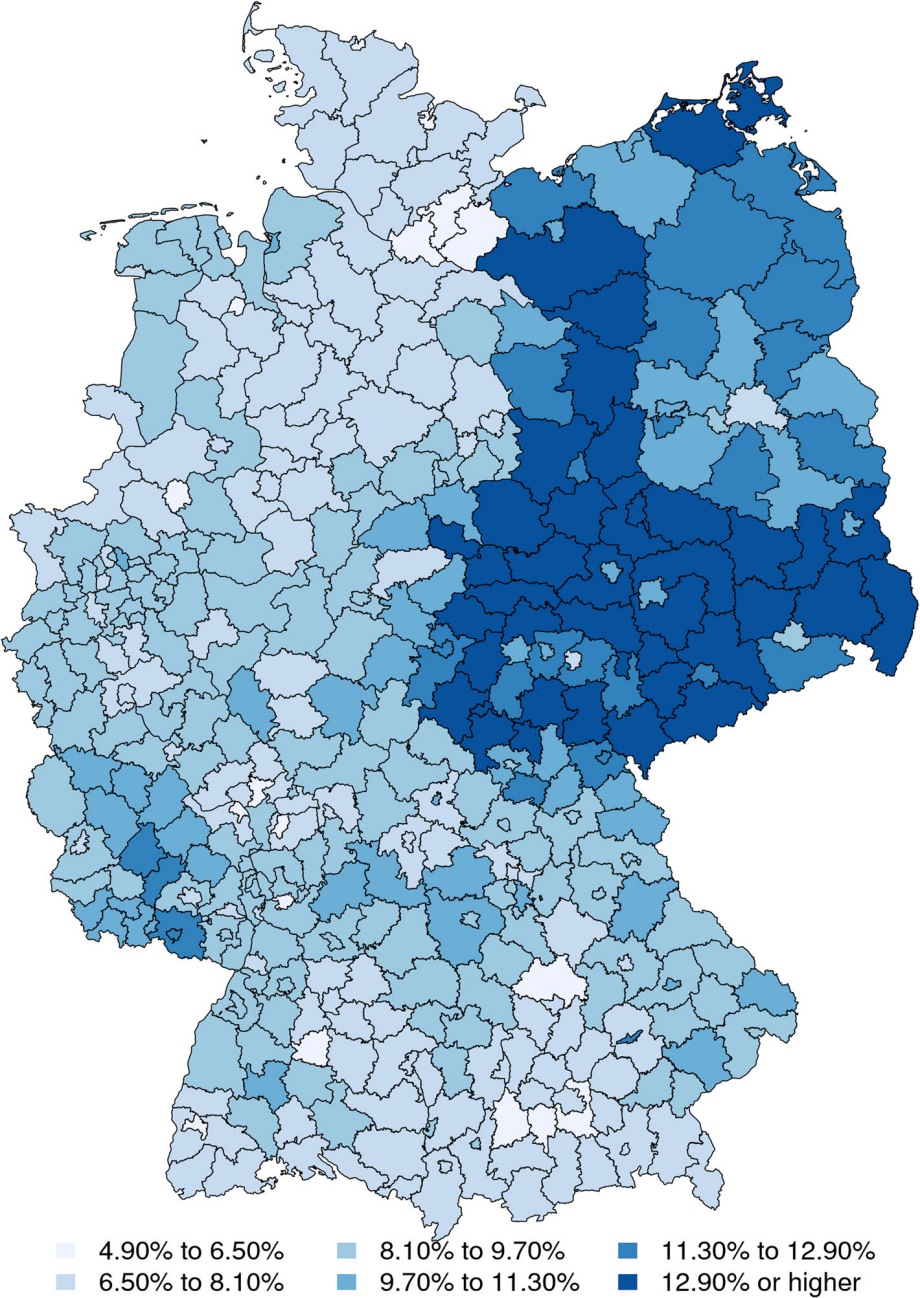
Estimated district level prevalence

Figure 3 displays the estimated diabetes prevalence of the national population on the district level. A high diabetes morbidity can be seen in the former territory of the German Democratic Republic (eastern Germany). This is potentially due to the relatively high level of economic deprivation in these regions, given the fact that diabetes has been frequently associated with low economic status in past studies. Further, eastern Germany is inhabited by large fractions of elderly. Younger citizens tend to move to areas with more economic activity and better job perspectives. Since diabetes morbidity also increases significantly with age, it is expected that the highest prevalence is located in this part of the country. The region with relatively low diabetes prevalence in the northeast of Germany is Berlin. It likely has a smaller morbidity because it is the nation’s capital and thus attracts a large number of younger citizens.

**Fig. 4:**
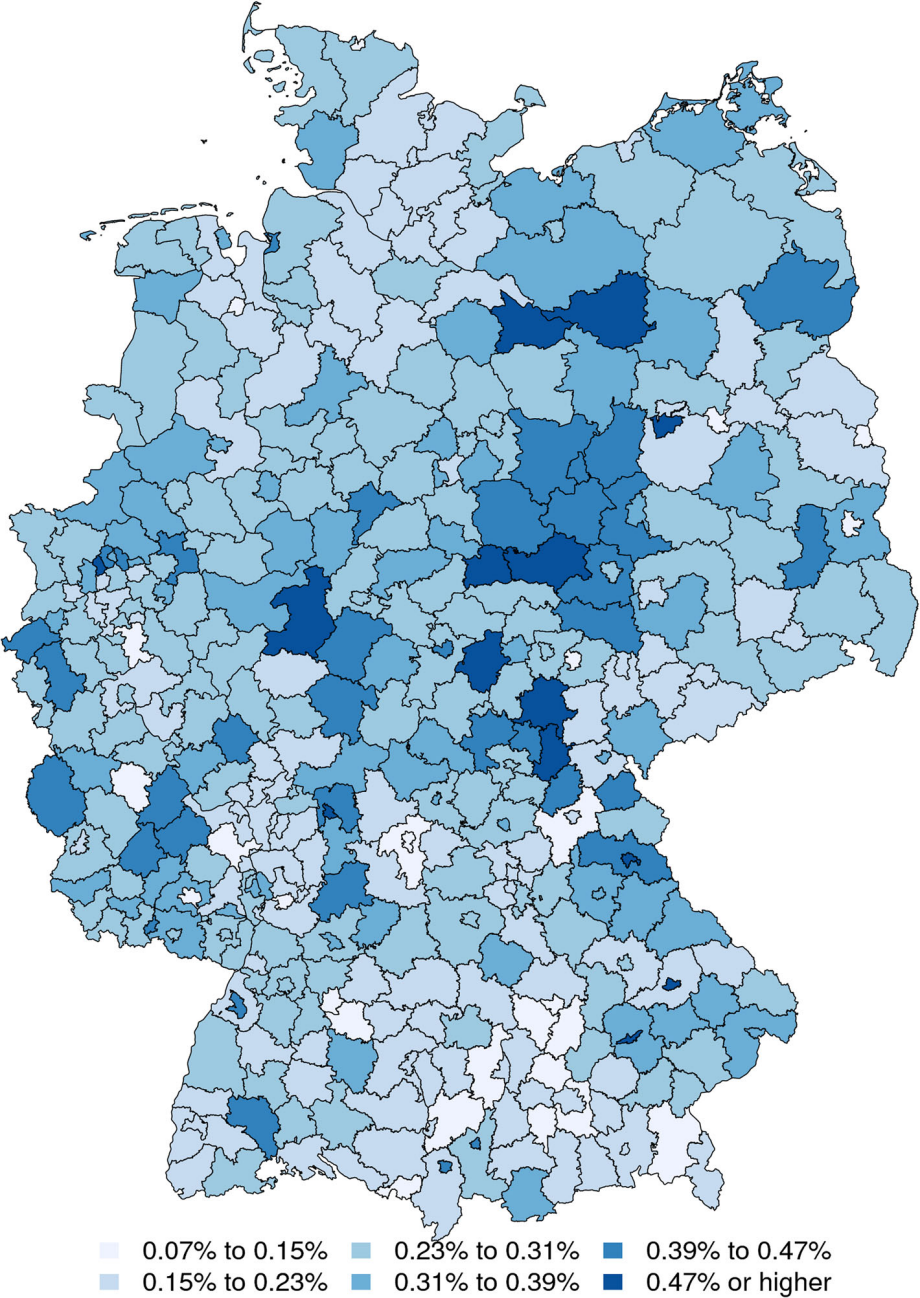
Estimated district prevalence, 34 and younger

Figure 4 shows the estimated diabetes prevalence of the national population on the district level with respect to age group I. As can be seen, the morbidity differences between eastern and western Germany are far less evident than in Fig. 3. While there is still a slight northeast-southwest gradient in the map, the overall distribution is much more homogeneous. This implies that the differences in local diabetes morbidity are partially explainable by regional demography.

### Validation

The presented results are now validated using external information on diabetes mellitus type 2 prevalence in Germany from past studies. However, note that an actual numerical comparison of our estimates to existing data is mainly possible on the national level due to a lack of reliable regional figures. For regional results, only general patterns can be compared convincingly, as external regional prevalence estimates are usually obtained from survey data with few local observations and thus subject to high uncertainty.

The estimated nationwide prevalence is compared to administrative diabetes mellitus type 2 records published by [24]. They derived prevalence figures from ambulatory physician reimbursement data for all statutory HI members in Germany for the years 2009 to 2015. For 2014, they estimated an overall prevalence rate of 9.37% for the statutory HI member population. As our estimates refer to the national population, including statutory HI as well as private HI members, and private HI members are known to have significantly lower diabetes morbidity relative to the statutory HI average [15], the national population prevalence has to be further adjusted to estimate the statutory HI prevalence (see the Appendix 2 for details). The resulting prevalence of the statutory HI member population based on our model is 9.36% with a 95% confidence interval of [8.68%, 10.27%]. Accordingly, the estimated statutory diabetes prevalence is consistent with the administrative statutory prevalence records.

Regarding the adjustment behaviour of the model, Figs. 1 and 2 suggest that the prevalence estimates are mainly obtained from negative adjustments of the original AOK member prevalence. This is consistent with the results of [10, 15, 16], who found that the AOK has the highest diabetes prevalence among all German HIs. This consistency and the consistency in terms of the administrative prevalence records make a strong case for the effectiveness of the proposed methodology. Regarding the estimation of regional prevalence patterns, the estimated diabetes distribution shows the northeast-southwest gradient with large morbidity differences between eastern and western Germany. Corresponding findings have also been obtained from administrative records by [24] and from survey data by [17, 25].

We further compare our estimated diabetes distribution numerically to survey-based estimates published by [25]. The corresponding figures are on the federal state level and referenced by sex. In the following, we focus on the male population. Corresponding estimates are obtained from the nationwide health survey “Gesundheit in Deutschland aktuell (GEDA)”. It encloses roughly 10,000 male participants and was conducted in 2014 and 2015. It covers both statutory and private HI members (see [26] for more details). However, in the light of the issues associated with regional prevalence estimates from survey data, the validity of the subsequent comparison is very limited. Further, the authors used a different profile to identify diabetes mellitus type 2 (12-month prevalence). Therefore, we only compare our model estimates to survey-based 95% confidence intervals stated in this publication. Given the limited validity of the comparison, this should not be overemphasized.

Table 1 shows our prevalence estimates for the male population on the federal state level. The model estimates are compared to survey-based confidence intervals of [25] obtained from GEDA. It can be seen that the model estimates coincide with the survey-based intervals for 14 out of 16 federal states. Accordingly, the model results are consistent with the survey-based results for the majority of regions. However, they do not coincide with the survey-based results for Thuringia and Mecklenburg-Western Pomerania. Here, it is important to mention that when looking at other studies, the survey-based results obtained from GEDA for these two federal states are implausibly low. The results from the administrative records published by [24], which cover millions of statutory HI members, display Thuringia and Mecklenburg-Western Pomerania among the highest in terms of diabetes mellitus type 2 prevalence, which is consistent with our model estimates. Although the administrative records refer only to statutory HI members, this discrepancy to the survey-based GEDA results cannot be caused exclusively by the not-covered private HI members. Accordingly, our model estimates for Thuringia and Mecklenburg-Western Pomerania are more plausible.

**Table 1:** Comparison of federal state prevalence, male population

Federal state	Model estimate (%)	GEDA 95% confidence interval (%)
Schleswig-Holstein	06.72	06.2–10.7
Hamburg	04.90	04.3–08.7
Bremen	08.16	04.8–13.4
Lower Saxony	07.98	06.0–09.5
North Rhine-Westphalia	08.16	07.1–10.2
Hesse	08.18	06.8–10.2
Rhineland-Palatinate	09.03	08.9–12.5
Baden-Württemberg	08.21	05.6-08.7
Bavaria	08.32	06.5–09.5
Berlin	06.97	04.8–09.1
Saarland	10.17	07.8–14.2
Mecklenburg-Western Pomerania	12.02	04.6–10.0
Brandenburg	11.72	09.4–16.5
Saxony-Anhalt	13.78	12.2–22.4
Thuringia	12.64	07.0–09.6
Saxony	12.67	07.8–17.1

## Discussion

The comparison of our model estimates with results from past studies suggests that regional prevalence estimation can be performed accurately from health insurance records despite fund-specific selectivity. However, the presented methodology has some limitations that are discussed hereafter. Firstly, it is mainly applicable to diseases that are either very common or have closely-related comorbidities that can be retrieved from inpatient diagnosis frequencies. Within this study, we considered diabetes mellitus type 2, which is both a common medical condition and also known to have comorbidities that are visible in hospital data. In a joint research project with the AOK, we further obtained comparably good results for various other diseases, e.g. dementia or myocardial infarction. However, the method showed poor performance for rare diseases without closely-related comorbidities in hospital data, such as multiple sclerosis.

In some cases, if a disease has no strong predictors within inpatient diagnosis frequencies, other routine data sources for prevalence adjustment may be suitable instead. A potential alternative could be records on ambulatory treatments or prescribed medicine. In Germany, corresponding data is gathered by the Association of Statutory Health Insurance Physicians. However, despite being generally of interest for prevalence adjustment, these records enclose information on statutory HI members only and are therefore not suitable for statistical inference regarding private HI members. Further, the data is at least partially only available at higher levels of aggregation. The usage of auxiliary data with a higher aggregation level than the desired prevalence estimates requires the fund-specific selectivity to be sufficiently adjustable on the higher level. Selectivity on smaller scales cannot be accounted for. Accordingly, the usage of these alternative data sources for prevalence estimation as presented strongly depends on the nature of the fund-specific selectivity and the target of inference.

Another situation that may be problematic for the methodology is when the comorbidity variables selected for prevalence adjustment show regional morbidity patterns that do not exist within the actual distribution of the disease of interest. If these patterns are strongly evident over all predictors, then a prevalence adjustment would lead to false implications regarding the regional prevalence to be estimated. An example could be knee replacement, for which arthrosis is the corresponding diagnosis.

A further limitation that was already mentioned in the “Methods” section is the cell size required for prevalence estimation. For simplicity, we demanded that the number of members per cell is not zero. This ensures that the functional relation between the diagnosis of interest and the auxiliary data is extracted from all cells for which prevalence estimates are desired. Note that this condition simplifies capturing morbidity patterns on local levels, but is technically not essential when making further homogeneity assumptions. However, in a classical estimation framework also, cells with a few members would be problematic, as they would lead to high variance in prevalence estimates. But as our approach marks a special case of small area estimation [21], the presented methodology is able to account for such cases. By calculating the conditional expectation of the cell prevalence given the inpatient diagnosis frequencies, we borrow strength from other cells and stabilize estimation. In fact, by extrapolating the conditional expectation, it is even possible to produce prevalence estimates for cells with zero members, as long as the auxiliary data has sufficient explanatory power, and the number of empty cells is not too large. If there is a substantial share of empty cells within a region, then the proposed multi-level model collapses due to zero inflation. In that case, other model types have to be considered (e.g. zero-inflated Poisson models).

Nevertheless, the proposed adjustment of HI member records for regional prevalence estimation holds a variety of advantages over survey-based methods. The first advantage is the level of regional detail for which prevalence estimates can be obtained. Model-based estimation methods are known to be much more stable relative to design-based methods for small aggregates [21]. Accordingly, even highly referenced prevalence estimates can be obtained with acceptable variance levels. The second advantage is a significant reduction in survey costs. With the proposed methodology, regional prevalence estimation does not require cost-intensive extended surveys. Instead, it is sufficient to use routine data that is collected automatically. The third advantage is the richness of HI records in terms of analysis possibilities. The variety of health-related information collected by HI funds allows for the investigation of many health-related research questions. The fourth advantage is privacy protection. The approach does not require micro-level data unlike many survey-based methods, which is particularly sensitive in the health context.

A remaining question is whether health insurance records can also be adjusted for other purposes than regional prevalence estimation. Generally, it seems plausible that the proposed methodology could be applied to different forms of medical research, e.g. clinical studies. The inclusion of routine data for benchmarking as presented could enhance propensity score matching that is often used in experiments. However, this is beyond the scope of this paper and thus subject to further research.

## Conclusions

A methodology to perform regional prevalence estimation from health insurance records despite fund-specific selectivity was proposed. The morbidity patterns of an individual health insurance fund were adjusted by conditioning the company-specific disease distribution on inpatient diagnosis frequencies. Using a multi-level linear mixed model, the conditional expectation was extrapolated using DRG Statistics data as a benchmark. An application was provided on the example of diabetes mellitus type 2 prevalence in Germany. The model managed to estimate age and sex referenced prevalence for all German districts. It was further able to reproduce available administrative prevalence figures with good accuracy. The approach contributes to future research and policymaking, as it allows regional prevalence estimation in great detail while reducing response burden and survey costs.

## Data Availability

The health insurance data we used are subject to confidentiality under national legislation. Hence, we are not allowed to provide them. All computations are based on aggregate data rather than individual data and were performed within a safe computer lab of the Scientific Institute of the AOK (WIdO), Berlin.

## Appendix 1

### Identification of AOK members with diabetes mellitus type 2

In the following, it is described how AOK members with diabetes mellitus type 2 were identified in the corresponding health insurance records. The identification was based on intersectoral data sets. Patients with ambulatory or inpatient diabetes diagnosis (ICD E10 - E14) are considered type-2-diabetes patients, if at least one of the following conditions during the respective calendar year is met:

- Patients have no insulin prescription (ATC code A10A)
- Patients have prescriptions of other antidiabetics excl. metformin (ATC code A10B or A10X excl. A10BA02)
- Inpatient with main diagnosis of only type-2-diabetes (ICD E11, no ICD E10 and no ICD E13)
- Participation in type-2-diabetes disease management programme DMP
- Ambulatory and inpatient secondary diagnoses—unambiguous cases (only ICD E11 diagnoses, no ICD E10 and no ICD E13)
- Ambulatory and inpatient secondary diagnoses—relative frequency with majority of ICD E11 diagnoses and number of cases with ICD E11 diagnoses ≥ other cases (ICD E10, ICD E13) + 2.

For the ICD codes, the international statistical classification of diseases and related health problems, German modification (ICD-10-GM) was used. ATC corresponds to the German anatomical therapeutic chemical classification.

## Appendix 2

### Prevalence estimation with respect to statutory HI members

In the following, it is described how the prevalence estimates obtained from model (4), that refer to both statutory HI and private HI members, are adjusted in order to quantify statutory HI member prevalences only. This is done in order to compare the model estimates to the results from [24], which refer exclusively to statutory HI members. Note that the records of the DRG statistic cannot be separated in statutory HI and private HI members. This is why a different adjustment is required. The adjustment is performed on the regional level using AOK records, national population demographic data obtained from [19], statutory HI member population demographic data obtained from [27] and epidemiologic information published by [15].

*Step 1: Quantifying the regional private HI members*

Let *N*_*r*_ denote the number of individuals (both statutory HI and private HI) in region *r* known from [19]; let ${N}_r^{\mathrm{SHI}}$ be the number of statutory HI members obtained from [27]. The number of PHI members in *r* is then given by:

$$ {N}_r^{\mathrm{PHI}}={N}_r-{N}_r^{\mathrm{SHI}}. $$

*Step 2: Estimating the regional private HI prevalence*

In [15], the diabetes mellitus prevalence of several HI funds were compared. They used survey data from 2004 to 2008 (total of 15089 participants) to estimate prevalence ratios between funds while controlling for several confounding variables. They also provided a ratio estimate of the crude AOK prevalence relative to the non-standardized overall private HI prevalence. Let this estimate be denoted by $\hat{\alpha}$. Assuming the ratio describes the prevalence relation between AOK and private HI members for all regions in expectation, it can be used to estimate the regional private HI prevalence using the known regional AOK prevalence ${y}_r^{\mathrm{AOK}}$:

$$ {\hat{y}}_r^{\mathrm{PHI}}=\frac{y_r^{\mathrm{AOK}}{N}_r^{\mathrm{PHI}}}{N_r^{\mathrm{AOK}}\hat{\alpha}}. $$

*Step 3: Estimating the regional statutory HI prevalence*

At this stage, the regional statutory HI prevalence can be estimated from the model predictions ${\hat{y}}_r$ and the private HI estimates ${\hat{y}}_r^{\mathrm{PHI}}$ obtained from the upper adjustment:

$$ {\hat{y}}_r^{\mathrm{SHI}}={\hat{y}}_r-{\hat{y}}_r^{\mathrm{PHI}}. $$

The estimated statutory HI prevalence rate is then obtained after dividing ${\hat{y}}_r^{\mathrm{SHI}}$ by the known ${N}_r^{\mathrm{SHI}}$. The estimates are concluded to be valid, if

$$ \frac{\sum_{r=1}^R{\hat{y}}_r^{\mathrm{SHI}}}{\sum_{r=1}^R{N}_r^{\mathrm{SHI}}}\approx \frac{y_{\mathrm{Ref}}^{\mathrm{SHI}}}{\sum_{r=1}^R{N}_r^{\mathrm{SHI}}}, $$

where ${y}_{\mathrm{Ref}}^{\mathrm{SHI}}$ denotes the reference prevalence. Note that only a comparison over all *R* regions (not the individual regions) is valid, as the estimated ratio $\hat{\alpha}$ is not spatially dividable.

## Abbreviations

AOK: German Public Health Insurance Company
DRG: Diagnosis-Related Group Statistics
HI: Health insurance

## References

[1] Mendez-Luck C, Yu H, Meng YY (2007). Estimating health conditions for small areas: asthma symptom prevalence for state legislative districts. Health Serv Res.

[2] Branscum A, Hanson T, Gardner I (2008). Bayesian non-parametric models for regional prevalence estimation. J Appl Stat.

[3] Manzi G, Spiegelhalter DJ, Turner R (2011). Modeling bias in combining small area prevalence estimates from multiple surveys. J R Stat Soc Ser A Stat Soc.

[4] Stern S (2014). Estimating local prevalence of mental health problems. Health Serv Outcome Res Methodol.

[5] Zhang X, Holt JB, Wheaton AG (2014). Multilevel regression and poststratification for small-area estimation of population health outcomes: a case study of chronic obstructive pulmonary disease prevalence using the behavioral risk factor surveillance system. Am J Epidemiol.

[6] European Parliament and the Council of the European Union. Regulation (eu) no. 282/2014 of the european parliament and the council of 11 march 2014 on the establishment of a third programme for the union’s action in the field of health (2014-2020). Off J Eur Union. 2014;L86(1) URL https://data.europa.eu/eli/reg/2014/282/oj.

[7] Subramanian SV, Kubzansky L, Berkman L (2006). Neighborhood effects on the self-rated health of elders: uncovering the relative importance of structural and service-related neighbourhood environments. J Gerontol B Psychol Sci Soc Sci.

[8] Ellaway A, Benzeval M, Green MJ (2012). Getting sicker quicker: does living in a more deprived neighbourhood mean your health deteriorates faster?. Health Place.

[9] Hoffmann F, Koller D (2017). Different regions, differently insured populations? Sociodemographic and health-related differences between insurance funds. Gesundheitswesen.

[10] Hoffmann F, Icks A (2012). Diabetes “epidemic” in Germany? A critical look at health insurance data sources. Exp Clin Endocrinol Diabetes.

[11] Jaunzeme J, Eberhard S, Geyer S (2013). Wie “repräsentativ” sind GKV-Daten?. Bundesgesundheitsblatt.

[12] Grunow M, Nuscheler R (2014). Public and private health insurance in Germany: the ignored risk selection problem. Health Econ.

[13] Hoffmann F, Bachmann C (2014). Unterschiede in den soziodemografischen Merkmalen, der Gesundheit und Inanspruchnahme bei Kindern und Jugendlichen nach ihrer Krankenkassenzugehörigkeit. Bundesgesundheitsblatt.

[14] Bundesministerium für Arbeit und Soziales. Referentenentwurf zur Sozialversicherungsrechengrößenverordnung 2017. Online; 2016. URL https://www.bmas.de/DE/Presse/Meldungen/2016/referentenentwurf-zur-sozialversicherungs-rechengroessenverordnung.html. Retrieved on August 1st, 2019.

[15] Hoffmann F, Icks A (2011). Diabetes prevalence based on health insurance claims: large differences between companies. Diabet Med.

[16] Hoffmann F, Icks A (2012). Structural differences between health insurance funds and their impact on health services research: results from the Bertelsmann health-care monitor. Gesundheitswesen.

[17] Schipf S, Werner A, Tamayo T (2012). Regional differences in the prevalence of known type 2 diabetes mellitus in 45-74 years old individuals: results from six population-based studies in Germany (DIAB-CORE Consortium). Diabet Med.

[18] Statistisches Bundesamt. Fallpauschalenbezogene Krankenhausstatistik (DRG-Statistik) Diagnosen, Prozeduren, Fallpauschalen und Case Mix der vollstationären Patientinnen und Patienten in Krankenhäusern. Online; 2015. URL https://www.destatis.de/GPStatistik/servlets/MCRFileNodeServlet/2120640147004_akt11012016.pd/2120640147004_akt11012016.pdf. Retrieved on August 1st, 2019.

[19] Statistisches Bundesamt. Bevölkerungsstand. Online, 2016. URL https://www.destatis.de/DE/Themen/Gesellschaft-Umwelt/Bevoelkerung/Bevoelkerungsstand/Tabellen/altersgruppen-familienstand-zensus.html. Retrieved on on September 26th, 2016.

[20] Pinheiro José C., Bates Douglas M. (2000). Mixed-Effects Models in Sand S-PLUS.

[21] Rao JNK, Molina I. Small Area Estimation, Wiley Series in Survey Methodology. 2nd ed: Wiley; 2015.

[22] Vaida F, Blanchard S (2005). Conditional akaike information for mixed-effects models. Biometrika.

[23] Morris JS (2002). The blups are not “best” when it comes to bootstrapping. Stat Probab Lett.

[24] Goffrier B, Schulz M and Bätzing-Feigenbaum J. Administrative Prävalenzen des Diabetes Mellitus von 2009 bis 2015. Technical report, Zentralinstitut für die kassenärztliche Versorgung in Deutschland, 2017. Report No. 17/03. 10.20364/VA-17.03.

[25] Heidemann C, Kuhnert R, Born S (2017). 12-Monatsprävalenz des bekannten Diabetes Mellitus in Deutschland. J Health Monit.

[26] Saß AC, Lange C, Finger JD (2017). “Gesundheit in Deutschland aktuell” – Neue Daten für Deutschland und Europa. Hintergrund und Studienmethodik von GEDA 2014/2015-EHIS. J Health Monit.

[27] Bundesministerium für Gesundheit. KM 6-Statistik (gesetzliche Krankenversicherung: Versicherte). Online; 2017. URL https://www.bundesgesundheitsministerium.de/themen/krankenversicherung/zahlen-und-fakten-zur-krankenversicherung/mitglieder-und-versicherte.html. Retrieved on August 1st, 2019.

